# Infection with Hepatitis C Virus among HIV-Infected Pregnant Women in Thailand

**DOI:** 10.1155/2008/840948

**Published:** 2009-01-27

**Authors:** Denise J. Jamieson, Natapakwa Skunodom, Thanyanan Chaowanachan, Anuvat Roongpisuthipong, William A. Bower, Tawee Chotpitayasunondh, Wendy Bhanich Supapol, Wendi L. Kuhnert, Wimol Siriwasin, Jeffrey Wiener, Sanay Chearskul, Michelle S. McConnell, Nathan Shaffer

**Affiliations:** ^1^Division of Reproductive Health, Centers for Disease Control and Prevention, Atlanta, GA 30341-3717, USA; ^2^Thailand MOPH-U.S. CDC Collaboration, CDC, Nonthaburi 11000, Thailand; ^3^Faculty of Medicine Siriraj Hospital, Mahidol Univeristy, Siriraj, Bangkoknoi, Bangkok 10700, Thailand; ^4^Division of Viral Hepatitis, Centers for Disease Control and Prevention, Atlanta, GA 30333, USA; ^5^Queen Sirikit National Institute of Child Health, Department of Medical Services, Ministry of Public Health, Bangkok 10400, Thailand; ^6^Department of Public Health Sciences, Faculty of Medicine, University of Toronto, Toronto, Canada ON M5S 1A8; ^7^Department of Medical Services, Rajavithi Hospital, Ministry of Public Health (MOPH), Bangkok 10400, Thailand; ^8^Global AIDS Program, Centers for Disease Control and Prevention, Atlanta, GA 30333, USA

## Abstract

*Objective*. The purpose of this study was to describe the epidemiology of coinfection with hepatitis C virus (HCV) and HIV among a cohort of pregnant Thai women. *Methods*. Samples from 1771 pregnant women enrolled in three vertical transmission of HIV studies in Bangkok, Thailand, were tested for HCV. *Results*. Among HIV-infected pregnant women, HCV seroprevelance was 3.8% and the active HCV infection rate was 3.0%. Among HIV-uninfected pregnant women, 0.3% were HCV-infected. Intravenous drug use by the woman was the factor most strongly associated with HCV seropositivity. Among 48 infants tested for HCV who were born to HIV/HCV coinfected women, two infants were HCV infected for an HCV transmission rate of 4.2% (95% 0.51–14.25%). *Conclusions*. HCV seroprevalence and perinatal transmission rates were low among this Thai cohort of HIV-infected pregnant women.

## 1. INTRODUCTION

Worldwide, the hepatitis C virus (HCV) seroprevalence rate among pregnant women is approximately 1% [1]. This is similar to the 1.6% prevalence of HCV antibody in the general population in the United States [2]. Among HIV-infected pregnant women, much higher prevalences of HCV positivity have been reported, ranging from as high as 30 to 50% in some settings [3, 4], particularly in populations with high rates of injection drug use.

Although the perinatal transmission rate of HCV is estimated to be less than 5% among HIV-uninfected women, it is generally higher in HIV-infected women [1]. Not all studies, however, have found increased HCV transmission rates among HIV-infected women [5] and a wide range of estimates of the risk of vertical transmission of HCV among coinfected women has been reported with wide geographic variation [6, 7].

The purpose of this study was to describe the epidemiology of coinfection with hepatitis C virus and HIV among a cohort of pregnant Thai women.

## 2. MATERIALS AND METHODS

The current study population includes 1771 women previously enrolled in three vertical transmission studies in Bangkok, Thailand [8–10] who had specimens available for HCV testing. From the first study (peri-1), 342 HIV-uninfected women and 293 HIV-infected women are included. From the second (peri-2) and third studies (peri-3), 391 and 745 HIV-infected women are included, respectively. Women in these studies were either enrolled during antenatal care (peri-1 and 2) or at delivery (peri-3). All children born to HIV-infected women were followed for 4–18 months and their mothers did not breastfeed. No follow-up information is available for infants born to HIV-uninfected women in peri-1. Peri-1 was an observational prospective cohort study without treatment interventions, peri-2 was a randomized placebo-controlled clinical trial assessing the efficacy of short-course zidovudine prophylaxis antenatally and intrapartum, and peri-3 was an observational study of short-course zidovudine and single-dose nevirapine propylaxis. All three studies were conducted jointly by the Thailand Ministry of Public Health and the U.S. Centers for Disease Control and Prevention (CDC) at two large Bangkok hospitals from 1992 to 2004. The three perinatal studies (peri-1,2,3) and this current retrospective laboratory study were all approved by the institutional review boards at the CDC in Atlanta and the Ethical Review Committees for Research in Human Subjects at the Thailand Ministry of Public Health and Siriraj Hospital in Bangkok.

In the original studies, specimens were collected and tested for HIV, HIV viral load, CD4 count as previously described [8, 9]. Stored plasma specimens from pregnancy or delivery were screened for antibodies to HCV (anti-HCV) using enzyme immunoassay (EIA; Abbott Murex version 4.0, Abbott Laboratories, Abbott Park, Ill USA). All positive EIAs were tested with qualitative reverse transcriptase polymerase chain reaction (RT-PCR; Ampliscreen, Roche Diagnostic Systems, Branchburg, NJ, USA). If the qualitative RT-PCR was negative, the plasma specimen was retested with recombinant immunoblot assay (RIBA 3.0, Chiron Corporation, Emeryville, Calif, USA). All women who were EIA positive and either qualitative RT-PCR or RIBA positive are considered HCV infected (either current or past); all other women are considered HCV uninfected. All specimens with detectable virus were tested with quantitative PCR (COBAS Amplicor HCV Monitor Test, version 2.0, Roche Diagnostic Systems) with a lower limit of detection of <600 copies/mL. All women with detectable virus are considered to have active HCV infection. Specimens with detectable HCV were genotyped using the Trugene HCV 5′NC genotyping kit (Bayer HealthCare LLC, Berkeley, Calif, USA) and sequence analysis with the OpenGene DNA sequencing system. Results were then confirmed using sequence analysis (ABI PRISM 310 Genetic Analyzer, Applied Biosystems, Calif, USA) methods. In 8 cases where the results from the two techniques were discordant, the 715 nt E1-E2 region at position 883-1597 nt was directly sequenced.

Stored plasma from infants born to women coinfected with HIV and HCV was also tested for HCV. An HCV-infected infant was defined as an infant who was anti-HCV positive (i.e., EIA-positive with confirmatory RIBA) at 18 months of age or older or who had positive HCV RNA on two occasions. Several serial samples from infants were tested, depending on availability of samples and testing results, since HCV infection often cannot be excluded in infants by one-time testing [11]. However, due to limited availability infant specimens, 18/48 (37.5%) of infants tested had testing at only one time point.

Statistical analyses were performed using SAS software version 9.1 (SAS Institute, Cary, NC, USA). Odds ratios with 95% confidence intervals were estimated using unconditional logistic regression, adjusting for the three perinatal studies. Ninety-five percent confidence intervals for HCV transmission rates were estimated using exact binomial methods.

## 3. RESULTS

There were low rates of intravenous drug use (1.5%) among the 1771 women included in this study (Table 1). A higher proportion of women reported several other risk factors for hepatitis C acquisition, including having an injection drug-using partner (10.2%) and ever having been a commercial sex worker (7.8%). HIV-infected women had moderately high CD4 counts at delivery (mean 428 cells/mm^3^); the mean viral load at delivery was 10 000 copies/mL.

Among 1429 HIV-infected pregnant women 62 (4.3%) were found to have HCV antibodies by EIA. Of those, 54 had positive confirmatory testing by either RT-PCR (*n* = 43) or by RIBA (*n* = 11). Thus, 3.8% (54/1429) of HIV-infected pregnant women were found to be coinfected with HCV and 3.0% (43/1429) had evidence of active infection. Among 342 HIV-uninfected women, 2 (0.6%) were found to have HCV antibodies by EIA. Of those, only one of the women had positive confirmatory testing by RT-PCR. Thus, 0.3% (1/342) of HIV-uninfected pregnant women were found to be infected with HCV.

Among the 54 HCV-seropositive women, 22 did not receive any antiretroviral prophylaxis, 22 received 4 weeks of antenatal zidovudine and intrapartum zidovudine, and 10 received 4 weeks of antenatal zidovudine and intrapartum zidovudine and nevirapine. Of the 43 HIV-infected women with quantifiable HCV, the serum levels of HCV RNA were as follows: 12 women with <100 000 copies/mL, 17 women with 100 000–850 000 copies/mL, and 13 women with >850 000 copies/mL. One woman who was HCV RNA-PCR positive on the qualitative assay had undetectable virus (<600 copies/mL) on the quantitative assay. The HIV-uninfected woman with quantifiable HCV had a serum level of HCV of 62 000 copies/mL. The HIV-infected women in later cohorts (peri-2 and peri-3) were more likely to be HCV infected (4.1 and 4.4%, resp.) compared with HIV-infected women in the earlier cohort (1.7%) (see Table 1). Among the 35 HCV-infected women with confirmed HCV genotyping results, 14 (40%) were genotype 3a, 14 (40%) were genotype 1a, 4 (11.4%) were genotype 1b, 2 (5.7%) were genotype 6a, and 1 (2.9%) was genotype 4a.

In unadjusted analyses, factors associated with pregnant women being HCV infected included more education, intravenous drug use, having a partner with a history of injection drug use, ever having been a commercial sex worker, and having received a blood transfusion. These risk factors remained significant when adjusting for perinatal study (Table 2). In a multivariate model adjusted for all covariates in Table 2, only three factors remained significant: intravenous drug use (adjusted odds ratio 70.5; 95% CI 24.8–201), having a partner with a history of injection drug use (adjusted odds ratio 3.4; 95% CI 1.5–7.4), and having received a blood transfusion (adjusted odds ratio 6.7; 95% CI 1.9–23.9). Since HIV-uninfected women were only included in peri-1, we included only women in peri-1 when estimating the odds of HCV infection by HIV status. This study did not find an association between HIV infection status and HCV infection (adjusted odds ratio 5.9; 95% CI 0.7–51.0).

For peri-1, all 5 infants born to HIV/HCV-infected women had samples tested for HCV RNA at 2, 4, and 6 months and no infants were found to be HCV infected. For peri-2, 12/16 infants had 6-month samples available which were tested for HCV RNA and 13/16 infants had 18-month samples available which were tested for HCV antibodies. All infants had testing from at least one time point and 10 infants were tested at more than one time point. From peri-2, one infant was HCV-RNA positive at 6 months; additional testing of a 4-month sample from this infant confirmed that this infant was HCV-RNA positive. In addition, three infants were anti-HCV positive by EIA at 18 months. However, only one of these infants had positive confirmatory testing by RIBA. For peri-3, 15 infants had serial testing at both 2 and 4 months and 27 infants had testing performed for at least one time point. No infants were found to be HCV infected.

In summary, among 48 infants tested for HCV who were born to HIV/HCV coinfected women, two infants were HCV infected for an HCV transmission rate of 4.2% (95% 0.51–14.25%). One of these infants was born to a woman who was infected with HCV genotype 3a and had an HCV viral load of 764 323 copies/mL and an HIV viral load of 2092 copies/mL. The mother of this infant received zidovudine for 4 weeks antenatally and during labor; this infant was HIV uninfected. The other infant was born to a woman who was infected with HCV genotype 1a and had an HCV viral load of >850 000 copies/mL and an HIV viral load of 20 342. The mother of this infant did not receive any antiretroviral prophylaxis; this infant was HIV infected.

The HIV vertical transmission rate among infants born to the 1429 HIV-infected women was 12.9% (Table 3). There was a 9.4% (5/53) HIV transmission rate among HCV-infected mothers and a 13.0% (170/1306) HIV transmission rate among HCV-uninfected mothers (*P* = .445). The odds of an infant being HIV infected did not differ significantly by the mother's HCV status neither in unadjusted analyses (odds ratio 0.70; 95% CI 0.27–1.77), nor in analyses adjusted for perinatal study (adjusted odds ratio 0.82; 95% CI 0.32–2.12).

## 4. CONCLUSIONS

HCV infection in pregnancy is emerging as an increasingly important issue. Due to improved HCV blood screening, mother-to-child transmission of HCV has now replaced transfusion-associated transmission as the predominant mode of spread in children [1]. In this retrospective analysis of more than 1700 pregnant women in Thailand, most of whom were HIV infected, we found relatively low hepatitis C seroprevalence rates. Among the HIV-infected pregnant women, HCV seroprevalence was 3.8% and active HCV infection was 3.0%. Among HIV-uninfected pregnant women, HCV seroprevalence was 0.3%. The low rate of injection drug use in the population (1.5%) likely accounts for this relatively low HCV seroprevalence, which is only slightly higher than many general populations of pregnant women who are HIV uninfected. (1) In a study conducted in Thailand from 1993-1994 among a convenience sample of 120 HIV-infected women, 6.7% were anti-HCV positive by EIA [12]. In another study in Thailand in 1991, 2.7% of 883 women admitted to the hospital with gynecologic abnormalities had HCV antibodies [13]. Among Thai women reporting injection drug use, higher HCV prevalence rates have been reported, with 15% of 200 female injection drug users HCV infected in a recent study [14].

The risk factors for HCV infection identified, which included more education, intravenous drug use, having a partner with a history of injection drug use, ever having been a commercial sex worker, and having received a blood transfusion, were similar to risk factors associated with HCV in prior studies [15]. Although the number of HIV-uninfected women was small in this study, we did not find HIV infection to be a significant risk factor for HCV infection. As expected, a history of injection drug use was the strongest predictor of HCV infection (adjusted OR 126; 95% CI 48.6–326), similar to other studies among blood donors which have also identified injection drug use as a strong risk factor for HCV infection [15]. There are six major HCV genotypes, which are numbered 1–6 and subtyped a, b, and c. Type 1b is the most common genotype worldwide. Types 1a and 3a, which were the predominant genotypes in the present study, are largely associated with injection drug use [16, 17].

In the United States, routine HCV screening is recommended for persons with certain high-risk characteristics (e.g., history of injection drug use or blood transfusion) and for children born to HCV-infected women [18]. However, routine screening is not recommended for pregnant women without other risk factors [19]. U.S. guidelines also recommend that HIV-infected persons be routinely screened for HCV [20]. In Thailand, although current national guidelines do not address routine HCV screening or hepatitis testing for HIV-infected patients, most physicians provide hepatitis testing for HIV-infected adults with symptoms or risk factors, and pediatric providers in tertiary care centers often test HIV-infected children who are at risk. Pregnant women in antenatal clinics are also routinely screened for hepatitis B, but not HCV. HCV treatment is available in Thailand at cost to patients or to patients with private health insurance.

The HCV perinatal transmission rate among infants born to HIV/HCV coinfected women in this study was 4.2%. However, due to the small number of HCV-infected women in our sample, the confidence interval was wide. The estimated frequency of HCV transmission in this current study is similar to those of two recent multicenter studies conducted among mostly HIV-uninfected women in the United States [21] and Europe [22] which reported transmission rates of 3.6% and 6.2%, respectively. Since HIV-infected women in this cohort had relatively high CD4 counts at delivery, it is not clear how generalizable these findings are to other cohorts of HIV-infected pregnant women. Although the published literature has shown a correlation of maternal HCV viral load and the risk of HCV vertical transmission [4, 23], there were too few HCV mother-to-child transmission in our study to draw conclusions. However, both HCV-infected infants born to HIV-infected women had high HCV viral loads.

## Figures and Tables

**Table 1:** Demographic and clinical characteristics of 1771 women enrolled in 3 vertical transmission HIV studies in Bangkok, Thailand, 1992–2004 (*n* (%)).

	*Peri-1 (HIV − )*(*n* = 342)	*Peri-1 (HIV+)*(*n* = 293)	*Peri-2*(*n* = 391)	*Peri-3*(*n* = 745)	*Total*(*n* = 1771)
HCV status					
* * Infected	1 (0.3)	5 (1.7)	16 (4.1)	33 (4.4)	55 (3.1)
* * Uninfected	341 (99.7)	288 (98.3)	375 (95.9)	712 (95.6)	1716 (96.9)
Education					
* * Primary or less	227 (66.4)	197 (67.2)	225 (57.5)	418 (56.1)	1067 (60.3)
* * Greater than primary	115 (33.6)	96 (32.8)	166 (42.5)	327 (43.9)	704 (39.8)
Marital status					
* * Married	179 (52.3)	174 (59.4)	185 (47.3)	248 (33.3)	786 (44.4)
* * Not married	163 (47.7)	119 (40.6)	206 (52.7)	497 (66.7)	985 (55.6)
Intravenous drug user (IDU)					
* * Yes	0	3 (1.0)	5 (1.3)	19 (2.6)	27 (1.5)
* * No	342 (100)	290 (99.0)	386 (98.7)	726 (97.5)	1744 (98.5)
Any partners IDU*					
* * Yes	5 (1.5)	20 (7.1)	38 (10.6)	102 (15.9)	165 (10.2)
* * No	335 (98.5)	260 (92.9)	321 (89.4)	539 (84.1)	1455 (89.8)
Ever a commercial sex worker*					
* * Yes	6 (1.8)	30 (10.2)	36 (9.3)	66 (8.9)	138 (7.8)
* * No	336 (98.3)	263 (89.8)	353 (90.8)	679 (91.1)	1631 (92.2)
Received transfusion (1985–89)*^+^					
* * Yes	5 (1.5)	3 (1.0)	10 (2.6)	15 (2.0)	33 (1.9)
* * No	334 (98.5)	290 (99.0)	380 (97.4)	730 (98.0)	1734 (98.1)
HIV subtype*					
* * E	—	273 (95.5)	318 (88.8)	559 (88.7)	1150 (90.3)
* * B	—	10 (3.5)	22 (6.2)	45 (7.1)	77 (6.0)
* * E/B	—	3 (1.1)	16 (4.5)	12 (1.9)	31 (2.4)
* * BR/MN/C	—	0	2 (0.6)	14 (2.2)	16 (1.3)
Delivery type*					
* * Cesarean	—	33 (11.3)	56 (14.3)	188 (25.2)	277 (19.4)
* * Vaginal	—	260 (88.7)	335 (85.7)	557 (74.8)	1152 (80.6)
Maternal age, years [mean (range)]	24.5 (15–40)	23.2 (14–40)	24.9 (17–39)	26.3 (15–48)	25.1 (14–48)
Age at 1st intercourse [mean (range)]	20.0 (13–32)	19.3 (11–35)	19.4 (12–32)	18.6 (7–44)	19.2 (7–44)
Gravidity [mean (range)]	1.8 (1–4)	1.6 (1–4)	1.8 (1–11)	2.1 (1–7)	1.9 (1–11)
Number of sex partners in past year [mean (range)]	1.0 (1-2)	1.8 (1–200)	1.1 (1–6)	6.9 (1–1428)	3.6 (1–1428)
Number of hours in labor [mean (range)]	—	7.4 (0–28)	10.6 (0–57.9)	14.4 (0–386)	12.0 (0–386)
Duration of membrane rupture, hours [mean (range)]	—	3.4 (0–96)	4.0 (0–39.5)	3.0 (0–148)	3.4 (0–148)
HIV viral load at delivery, log_10_copies/mL [mean (range)]	—	4.4 (2.3–6.2)	4.2 (2.3–5.9)	3.8 (2.3–6.0)	4.0 (2.3–6.2)
CD4 count at delivery, cells/mm^3^ [mean (range)]	—	471 (140–1180)	407 (11–1112)	421 (8–1608)	428 (8–1608)

*Sample size is decreased due to missing data. ^+^Thailand began screening blood for HIV in 1989.

**Table 2:** Odds of being HCV-infected among 1771 pregnant women enrolled in 3 vertical transmission HIV studies in Bangkok, Thailand, 1992–2004.

	*HCV-infection*				Adjusted*
	No.	%	(*n*)	OR (95% CI)	OR (95% CI)
Education					
* * Primary or less	1067	2.3	(24)	1.0	1.0
* * Greater than primary	704	4.4	(31)	2.00 (1.16–3.44)	1.81 (1.05–3.12)
Marital status					
* * Married	786	2.3	(18)	0.60 (0.34–1.06)	0.72 (0.40–1.29)
* * Not married	985	3.8	(37)	1.0	1.0
Intravenous drug user (IDU)					
* * Yes	27	74.1	(20)	140 (55.4–351)	126 (48.6–326)
* * No	1744	2.0	(35)	1.0	1.0
Any partners IDU^+^					
* * Yes	165	14.6	(24)	9.74 (5.42–17.5)	7.92 (4.34–14.4)
* * No	1455	1.7	(25)	1.0	1.0
Ever a commercial sex worker^+^					
* * Yes	138	6.5	(9)	2.40 (1.15–5.02)	2.17 (1.03–4.54)
* * No	1631	2.8	(46)	1.0	1.0
Received transfusion (1985–89)^+^					
* * Yes	33	12.1	(4)	4.65 (1.57–13.7)	4.22 (1.41–12.6)
* * No	1734	2.9	(50)	1.0	1.0
Maternal age, years	—	—	—	0.98 (0.93–1.04)	0.96 (0.90–1.02)
Gravidity	—	—	—	1.17 (0.93–1.47)	1.09 (0.86–1.38)
Number of sex partners in past year	—	—	—	1.00 (0.996–1.005)	1.00 (0.996–1.005)

*Adjusted for three perinatal studies. ^+^Sample size is decreased due to missing data.

**Table 3:** HIV/HCV status and clinical characteristics of infants born to 1429 HIV-infected women enrolled in 3 vertical transmission of HIV studies in Bangkok, Thailand, 1992–2004 (*n* (%)).

	*Peri-1*(*n* = 293)	*Peri-2*(*n* = 391)	*Peri-3*(*n* = 745)	*Total*
HIV status of infant*				
* * Infected	68 (24.4)	55 (14.4)	52 (7.5)	175 (12.9)
* * Uninfected	211 (75.6)	327 (85.6)	646 (92.6)	1184 (87.1)
HCV status of infant				
* * Infected	0	2 (0.5)	0	2 (0.1)
* * Uninfected	293 (100)	389 (99.5)	745 (100)	1427 (99.9)
Infant gender				
* * Male	136 (46.4)	207 (52.9)	365 (49.0)	708 (49.6)
* * Female	157 (53.6)	184 (47.1)	380 (51.0)	721 (50.5)
Birthweight				
* * < 2500 g	31 (10.6)	31 (7.9)	81 (10.9)	143 (10.0)
* * ≥ 2500 g	262 (89.4)	360 (92.1)	664 (89.1)	1286 (90.0)
Gestational age [mean (range)]	39.4 (28–44)	39.7 (35–45)	38.7 (31–44)	39.1 (28–45)

*Sample size is decreased due to missing data.
